# Evaluation of problem-based learning in medical students’ education

**Published:** 2014-01

**Authors:** MOHAMMAD HADI IMANIEH, SEYED MOHSEN DEHGHANI, AHMAD REZA SOBHANI, MAHMOOD HAGHIGHAT

**Affiliations:** 1 Gastroenterohepatology Research Center, Shiraz Transplant Research Center, Nemazee Teaching Hospital, Medical School, Shiraz University of Medical Sciences, Shiraz, Iran;; 2 Gastroenterohepatology Research Center, Shiraz University of Medical Sciences, Shiraz, Iran

**Keywords:** Medical students, Education, Teaching

## Abstract

**Introduction**: In traditional medical education systems much interest is placed on the cramming of basic and clinical facts without considering their applicability in the future professional career. The aim of this study is to evaluate a novice medical training method (problem-based learning) as compared to the contemporary teacher-based medical education or traditional methods.

**Methods**: Selection of the study subjects was done through simple sampling and according to the division of medical students introduced from Medical Faculty to the Pediatrics Department with no personal involvement. 120 medical students were assigned to 8 groups of 15 students each. For four months, 4 groups were trained with traditional method and 4 other groups underwent problem-based learning method on selected subject materials. In each method, a pre-course test at the beginning and a post-course test at the end of each course were given to each group. The questionnaire used in this study as the instrument was composed of 39 questions, 37 multiple choice questions and two short answer questions. Three professors of pediatric gastroenterologist took part in the training. Two of these professors were responsible for solving task training method. The third professor used traditional teacher-centered methodology to eliminate any possible bias. Scores obtained from these tests were analyzed using paired t-test and independent t-test. P-values of less than 0.05 were considered as significant.

**Results**: The scores of the students undergoing the traditional method were 14.70±3.03 and 21.20±4.07 in the first and second test, respectively. In problem-based learning, the scores were 15.82±3.29 in the first and 27.52±4.72 in the second test. There was a significant difference between the mean scores of post-course exams of the two groups (p=0.001), while no significant difference was observed between the mean scores of pre-course exams of the groups (p=0.550).

**Conclusion**: It may be concluded that problem-based learning method leads to a significant increase in learning and recalling output compared to the traditional method. Given the evolving medical education in the country's medical schools toward problem-based learning, it is suggested that the grounds be laid so that this change will take place based on thought, principles and problem solving.

## Introduction

There is growing concern among medical educators that conventional methods of teaching medical students (lecture-based curricula) neither encourage the appropriate qualities in students nor imparts a life-long respect for learning (1). Fundamental reforms in undergraduate medical education have been advocated for 100 years. In 1899, Sir William Osler realized that the complexity of medicine had already progressed beyond the ability of the teachers to teach everything that students would need to know. Osler recommended abolishing the lecture method of instruction and allowing students more time to study. He also emphasized the important role of teachers in helping students to observe and reason (2). In 1932, the Commission on Medical Education of the Association of American Colleges stated that medical education should develop sound habits as well as methods of independent study and thought; this will encourage the students to continue their self-education through life (3). This can be brought about only by eliminating some of the present rigidity and uniformity from medical education by reducing classroom over-crowding, and by adapting medical education to more closely meet the educational needs of students.

Undergraduate medical education, as with any other educational program, needs ongoing improvements to meet the changing demands of medical practice in the 21st century. Although the complexities of medical care have increased dramatically over the last century, the methods of teaching medicine have changed little. Teachers need to learn about the latest techniques and theories of medical education. Medical education should be given the same emphasis as research and patient care (4).

The aim of this study is to evaluate a novice medical training method (problem-based learning) in comparison to the contemporary teacher-based medical education using traditional methods. 

## Methods

In this research, the study sample included undergraduate medical students referred to the Pediatric Gastroenterology Ward of Nemazee Hospital during a four month period; they included 120 medical students, 60 as the test group and 60 as the control group. For determining the sample size through a pilot study, twenty medical students were evaluated. They were divided randomly into two test and control groups each with ten students and according to the average improvement score in individuals of the two groups, in level of error 0.05 and power 80%, sample size was calculated as 116 persons. Selection of the study subjects was done using simple sampling and according to the division of medical students introduced from Medical Faculty to the Pediatrics Department with no personal involvement or comments in special circumstances. Therefore, the selection of medical students as test or control groups was incidental. From the total study population, two students were excluded from the study because of absences in pre-test evaluation.

This study was a prospective interventional study. In general, the objectives of medical education were pediatric gastrointestinal diseases topics. The purpose was extracted from the book "General Practitioner in Iran, duties and training needs" in children’s digestive diseases (1).

Training needs theories were divided into four levels: A (known for at least practice), B (known to practice well), C (known to practice ideally) and D (those that know are not necessary). Three levels, A, B, and C were selected for this study. From different levels of general physician tasks in diagnosis of the diseases; level D1 (diagnosis at the level of history taking and physical examination is duty of the general physicians) and D2 (diagnosis at the level of simple and noninvasive para-clinical tests is duty of the general physicians) were selected. From different levels of general physician tasks in treatment of the diseases; level T0 (treatment is not the duty of the general physicians), T1 (initial treatment of the disease including symptomatic treatment is the duty of the general physicians) and T2 (final and advanced treatment of the disease is the duty of the general physicians) were selected. Totally 46 topics from pediatric gastrointestinal diseases including most common diseases and also less common diseases that a general practitioner needed to practice were selected.

The questionnaire used in this study was composed of 39 questions, 37 multiple choice questions and two short answer questions. Three professors of pediatric gastroenterology took part in the training. Two of these professors were responsible for solving task training method. A list of topics to be taught in each period was prepared. The third professor similarly taught through traditional teacher-centered method to eliminate any possible bias. A questionnaire was designed by MSc. students in the experimental group which helped to remove the bias.

In traditional training methods, the professor teaches the control group according to the admitted patients in the hospital or talks about some topics according to own discretion. All patients were referred by a student, and then the student discussed about the patient’s problems, clinical diagnostic approaches, and examined other possible diagnoses; the other students were trained in this way. Conferences were performed based on the fellowship and staff’s ideas and usually held in check by the speaker. The topics of discussion were on specialized subjects.

In planning and problem-based learning approach, students actively participated in the training sessions. In this case, the students were trained based on the selected topics. The student groups had subjective problems, possible clinical approaches to diagnosis and suggested possible diagnoses.

Each group was trained for 15 days. At the beginning of the course, an initial test was performed for all students (pre-test). After completing the course, the students had a secondary test (post-test). Two test questions were exactly the same. As mentioned earlier, the number of questions was 39, and 40 minute response time was also considered.

Scores obtained from these tests were assessed and analyzed in statistical software SPSS and statistical tests using paired t-test and independent t-test. 

## Results

Totally, 118 students completed the study, 59 in each groups. In the control group, pre-test mean score was 14.70±3.03 (range: 8-21) and in the test group, it was 15.82±3.29 (range: 9-25). The comparison between two groups regarding the mean score of the pre-test exam through independent t-test revealed that the test group was slightly better than the control one but their difference was not statistically significant (p=0.550).

 In the post-test evaluation, the mean score of the control group was 21.20±4.07 (range: 11-32) and in the test group was 27.52±4.72 (range: 15-37). The comparison between the post-test scores between the two groups by independent t-test showed that the test group has achieved better results than the control group which was statistically significant (p=0.001).

In the control group, the comparison between the pre-test and post-test scores by paired t-test showed that in the traditional learning method an average of 6.5 point was added which was statistically significant (p=0.001). In the test group, the comparison between the pre-test and post-test scores by paired t-test showed that 11.70 points were added to the pre-test exam score using problem-based learning method and this amount was significant (p= 0.001).

Table 1 shows the amount of increased correct responses regarding different types of questions in the two study groups. The correct answer rates were calculated based on the difference between the pre-test and post-test scores.

**Table 1:** The amount of increased correct responses regarding different types of questions in the two study groups

**Question type**	**Correct Responses (%)**	**Amount of increased correct responses in TLM (%)**	**Amount of increased correct responses in PBLM (%)**
A	52.29	14.2	29.6
B	53.91	23.51	8.57
C	29.31	19	7.25
T1	58.76	15.75	20.13
T2	50	20.65	21.36
T0	38	21.33	2.67
D1	64	14.17	33.67
D2	24	9	8.50

TLM; Traditional Learning Method, PBLM; Problem-based Learning Method

## Discussion

Traditional teaching separates the basic science segment from the clinical segment. In the conventional curriculum, teaching is tutor-centered and comprises large group lectures, tutorials, structured laboratory experience, and periodic tests of achievement (5). Problem-based learning is an instructional method in which students learn through facilitated problem solving. In problem-based learning, student learning focuses on a complex problem that does not have a single correct answer. Students work in collaborative groups to identify what they need to learn in order to solve a problem (6). Educational research indicates that this format of teaching is frequently unstructured, the acquisition of skills is left largely to chance and is subject to little quality control, students are inadequately monitored, and feedback is seldom given (7). Recent studies have reported the effects of problem-based learning during medical school training (7-9).

Hoffman et al. reported higher performance of problem-based learning graduates in the United States Medical Licensing Examinations (USMLE) (10). Finch reported that chiropody students at the Michener Institute for Applied Health Sciences who had experienced problem-based learning performed significantly better in tests of deeper understanding and the cognitive skills related to patient management, compared to the traditional cohort of students (11). In a study conducted among students studying special care in dentistry, a comparison of academic results showed that problem-based learning students scored better than those receiving conventional lectures (12).

Dolmans and Schmidt reported that in a problem-based learning curriculum as a major part of the curriculum that was delivered in problem-based learning mode, students become more experienced and better self-directed learners (13). In the present study, the perceived improvement in outcomes could be due to the fact that students are required to make a conscious effort to assume responsibility in their own learning through these active learning strategies.

Abraham and colleagues indicated that problem-based learning along with other active learning strategies employed in the curriculum right from first year resulted in the improvement of almost all the short-term outcomes. Furthermore, the adoption of problem-based learning demanded students to make a conscious effort to assume responsibility in their own learning, thereby resulting in better learning leading to better performance in the examinations (14).

According to the comparisons, we can conclude that the significant difference between pre-test and post-test mean scores of the control group (6.50) and the traditional teaching method in education has had a significant impact. The significant difference between pre-test and post-test mean scores of the test group (11.70) suggests that problem-based learning method has acceptable effects on the training program. Comparison between pre-test and post-test mean scores of the control and test groups shows that the mean scores for the test group was 5.20 point higher than that of the control group and because of this statistically significant difference (p=0.001), we can conclude that the planning and problem solving-based teaching method is more efficient i student education. Regarding type A questions that a general practitioner should know, about half of the students (52.29%) responded correctly, which was low. After training, in the traditional learning method 14.2% and in the problem based learning method 29.6% increase was seen in correct responses to this type of questions. The planned approach to topics that are necessary for a general practitioner offers a better education. In case of questions type B and C which were needed for general physician to practice well or ideally, the rates of correct answers were 53.91% and 29.31%, respectively; these amounts are acceptable for a general practitioner. The remarkable thing is that in traditional learning method an there was an increase of about 20% in correct responses, while in the problem-based learning method an increase of 8% was seen. So in the traditional education the topics in these levels were more considered. As to questions type D1 in which general physicians diagnosed the diseases according to the history taken and physical examination, about 64% of medical students answered correctly; this is acceptable but not sufficient for medical students. After training in the traditional method, 14.17% and in the problem solving method 33.67% increases in correct answers were found. Therefore a new training method is suitable for the easy detection, giving the students a better solution. Regarding questions type D2 in which general physicians should diagnose the diseases with simple and non-invasive para-clinical tests, the response rate was only 24% of all responses and it was very low. In both methods, an increase of about 9% was seen in these types of questions. So in this case, both methods were similar to those requiring further investigation. Questions type T1, in which the primary treatment is the duty of the general practitioner, response rate was 58.76%; this is somehow acceptable but insufficient. 15.75% increase in the traditional group and 20.13% increase in the problem solving group was observed. This suggests that a new way of teaching leads to better results. Teaching methods increased approximately 21% of the responses to questions type T2, so in this case no method was better than the other. 

## Conclusion

In planning and problem based learning method, the student will be actively involved in education; also the assessment of medical students and prospective doctors is done more efficiently and the disease will be more easily diagnosed and treated. 
